# Preoperative Low-Molecular-Weight Heparin Prophylaxis Associated with Increased Heparin Resistance Frequency in On-Pump Coronary Artery Bypass Graft Surgery

**DOI:** 10.1155/2019/4310407

**Published:** 2019-04-16

**Authors:** Onur Saydam, Mehmet Atay, Deniz Serefli, Suat Doganci, Ulas Kumabasar, Mustafa Yılmaz, Rıza Dogan, Metin Demircin

**Affiliations:** ^1^Tepecik Training and Research Hospital, Department of Cardiovascular Surgery, Izmir 35180, Turkey; ^2^Bakirkoy Dr. Sadi Konuk Training and Research Hospital, Department of Cardiovascular Surgery, Istanbul 34147, Turkey; ^3^Gulhane Training and Research Hospital, Department of Cardiovascular Surgery, Ankara 06010, Turkey; ^4^Hacettepe University Faculty of Medicine, Department of Cardiovascular Surgery, Ankara 06230, Turkey

## Abstract

**Background:**

Unfractionated heparin (UFH) and low-molecular-weight heparin (LMWH) are being used for preoperative management of critical coronary artery disease. However, preoperative UFH therapy may cause a reduction in antithrombin concentrations, leading to various degrees of heparin resistance (HR). The main purpose of this study is to investigate the effects of preoperative LMWH on HR during cardiopulmonary bypass (CPB).

**Methods:**

Data were retrospectively reviewed from adult patients that underwent on-pump coronary artery bypass graft (CABG) surgery. Four hundred fifty-seven patients underwent CABG, and 139 of them, who had isolated on-pump CABG, were included in the study. The heparin sensitivity index was calculated if activated clotting time levels were discovered below 400 seconds. Values less than 1.3 were accepted as HR.

**Results:**

Of 139 patients who underwent on-pump CABG, preoperative LMWH was administered in 59 patients (56.8%). Intraoperative HR occurred in 29 patients (20.9%). Patients who received preoperative LMWH had an increased risk of developing HR compared with patients who did not receive LMWH (odds ratio 4.8 and 95% confidence interval 1.7–13.5). CPB duration and aortic clamp duration were significantly longer in patients who developed intraoperative HR when compared to those in patients who did not develop HR.

**Conclusion:**

Preoperative treatment with LMWH may cause intraoperative HR. Corrective and preventive arrangements with close follow-up should be performed in this group of patients.

## 1. Introduction

Unfractionated heparin (UFH) is still the most reliable medication to prevent thrombosis and its catastrophic consequences during cardiopulmonary bypass (CPB). UFH indirectly inhibits coagulation by binding antithrombin (AT) [1]. UFH and AT complex bind thrombin, and UFH diverges from the complex. Afterward, thrombin-AT complex is consumed and eliminated by the reticuloendothelial system which leads to a decrease in AT levels. Preoperative UFH therapy may cause a reduction in AT concentrations, leading to various degrees of heparin resistance (HR) with this mechanism. CPB-associated HR is defined as the need for a higher dose of UFH than the standard dose to induce sufficient active coagulation time for CPB [1, 2]. HR is reported in up to 22% of patients undergoing CPB [2, 3].

AT deficiency is the primary mechanism of HR and can be either congenital or acquired. Acquired AT deficiency may associate with advanced liver disease, renal dysfunction, such as nephrotic syndrome, and malnutrition. Also, an upregulated hemostatic system such as disseminated intravascular coagulation, deep vein thrombosis, and endocarditis may lead to AT deficiency. However, one of the most common causes for acquired AT deficiency is a preoperative UFH treatment [4]. Although HR is more common in patients who receive UFH preoperatively, it may also be seen in patients treated with low-molecular-weight heparin (LMWH) [5, 6]. LMWH is frequently being used for preoperative prophylaxis of the coronary artery disease, especially in patients with critical stenosis [5–8]. Even though LMWH has fewer binding sites for AT, both LMWH and UFH require AT for their anticoagulant effect. As a result, UFH inactivates factor Xa and factor IIa equivalently, while LMWH mostly inactivates factor Xa [9–11].

The main purpose of this study is to investigate the effects of preoperative LMWH on HR in patients who underwent on-pump CABG surgery.

## 2. Methods

Data of adult patients that underwent on-pump CABG surgery in a single center from January 2012 to January 2014 were retrospectively collected by a file scanning method. The Institutional Ethics Committee of Hacettepe University approved the study protocol (GO 14/171-15). Among 457 patients who underwent CABG surgery, 139 patients who had isolated on-pump CABG surgery were included in the study. The rest of the patients, who had redo, off-pump, emergency CABG, or combined CABG and valve surgery, were excluded from the study.

To eliminate other factors that cause acquired AT deficiency in patients with malnutrition and liver or renal dysfunction including nephrotic syndrome, were excluded. Patients previously diagnosed with AT deficiency and personal or family history of a thromboembolic event were also excluded. We excluded 28 patients from the study with a personal or family history of a thromboembolic event.

### 2.1. Standard Preoperative and Intraoperative Anticoagulation Protocol

LMWH was administered preoperatively to patients with critical coronary artery disease (CAD) (significant left main CAD or left main equivalent CAD). Enoxaparin was used as an LMWH in all patients with a subcutaneous dosage of 0.01 cc/kg body weight. LMWH was administered twice daily for at least three preoperative days and was stopped 24 hours before surgery. After induction and intubation, baseline ACT was measured by Hemochron 801® device (Technidyne Corp., Edison, New Jersey, USA). Before cannulation, UFH with a dosage of 300 U/kg was administered through the right atrium and the ACT level was measured 5 minutes after UFH injection. CPB was commenced, when ACT level was over 400 seconds. When ACT level was below 400 seconds, additional UFH (150 U/kg) was applied. ACT was monitored every 20 minutes during CPB. After discontinuation of CPB, 1 mg of protamine was used to neutralize each 100 IU of heparin, and the ACT level was measured 5 minutes after the completion of protamine.

### 2.2. Heparin Sensitivity Index Calculation

After a retrospective data revision, for patients with an ACT level below 400 seconds after the first standard heparin dose, Heparin Sensitivity Index (HSI) was calculated by the following formula: ACT after UFH − baseline ACT/overall loading dose of heparin (IU/kg) [12]. Values less than 1.3 were accepted as HR.

### 2.3. Patient Population

**Figure 1:**
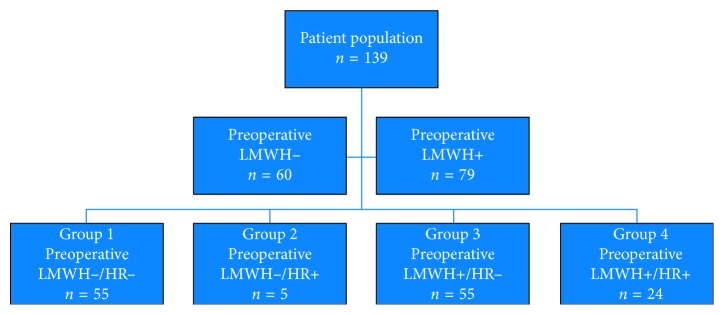
Patient population diagram. LMWH, low-molecular-weight heparin; HR, heparin resistance.

Patients were stratified into 4 groups according to the medical regimen and the presence of HR. Group 1 consisted of patients who did not receive LMWH before surgery and did not experience intraoperative HR (LMWH− / HR−). Group 2 consisted of patients that did not receive LMWH before surgery, but experienced intraoperative HR (LMWH−/HR+). Group 3 consisted of patients who received subcutaneous LMWH preoperatively, but did not experience intraoperative HR (LMWH+ / HR−). Group 4 consisted of patients who received subcutaneous LMWH preoperatively and experienced intraoperative HR (LMWH+/HR+) (Figure 1).

### 2.4. Study Parameters

Demographic data including age, gender, and weight, preoperative data including usage of acetylsalicylic acid (ASA) and clopidogrel, blood types, platelet counts, and perioperative baseline ACT levels, post-UFH and post-protamine sulfate ACT levels, CPB time, aortic clamp time, unit of fresh frozen plasma (FFP), and thrombocyte given were collected. Length of stay in intensive care unit and length of hospital stay were also evaluated.

### 2.5. Statistics

Sample size was calculated, and with this sample size, there is above 85% likelihood that this study will yield statistically significant results. To test the normality of distribution, the Shapiro–Wilk test was used. Continuous, normally distributed data are presented as mean and standard deviation. Nonnormally distributed interval data are also presented with the lowest and highest scores. Categorical data are presented as frequencies and percentages. To test differences for preoperative serum platelet levels, the Kruskal–Wallis test was used. Pair-wise comparisons of groups were performed with the Conover-Dunn test. Pearson's correlation test was performed to show a relation between lower platelet counts in patients who developed intraoperative HR. Logistic regression analysis was used to understand independent factors which affect HR. G ∗ Power 3 software was used to calculate achieved power for significant differences. A *P* value less than 0.05 was considered statistically significant, and a *β* type error was mentioned by <0.20 to represent achieved power.

## 3. Results

One hundred thirty-nine patients were evaluated. Patient demographics, clinical characteristics, and operative data are shown in Table 1. Group 1 (LMWH−/HR−) consisted of 55 patients, Group 2 (LMWH−/HR+) consisted of 5 patients, Group 3 (LMWH+/HR−) consisted of 55 patients, and Group 4 (LMWH+/HR+) consisted of 24 patients (Figure 1). There were no significant differences among the groups regarding age, gender, weight, preoperative usage of ASA and clopidogrel, blood type, length of stay in intensive care unit, and length of hospital stay.

Seventy-nine (56.8%) patients received LMWH preoperatively, and 29 (20.9%) patients experienced intraoperative HR. We found LMWH as an independent factor that affects HR and the rate of heparin resistance is increased 4.8 times in the patients who used LMWH before surgery (95% confidence interval 1.7–13.5, *P* value 0.003). There were significant differences for preoperative serum platelet levels. Those differences in platelet counts were between Group 2 (LMWH−/HR+) and Group 3 (LMWH+ / HR−) (168 ± 70 (×1000 *μ*L) vs 275 ± 85 (×1000 *μ*L), respectively, *p*=0.036) and between Group 3 (LMWH+ / HR−) and Group 4 (LMWH+/HR+) (275 ± 85 (×1000 *μ*L) vs 197 ± 75 (×1000 *μ*L), respectively *p*=0.001). There was a correlation between lower platelet counts with intraoperative HR (*p* < 0.001) (coefficient correlation −0.342). Basal levels of ACT were similar among the groups. Mean ACT levels following 5 minutes after the first UFH administration showed significant differences between the groups HR+ and HR− (450.69 ± 133.2 vs 633.58 ± 205.1, respectively, *p* < 0.001). ACT levels 5 minutes after protamine sulfate administration also showed a significant difference between groups with and without preoperative LMWH therapy (*p*=0.034). ACT levels 5 minutes after protamine sulfate administration showed significant differences between Group 2 (LMWH− / HR+) and Group 4 (LMWH+ / HR+) (154 ± 7 vs 133 ± 25, respectively, *p*=0.032) and Group 2 (LMWH−/HR+) and Group 3 (LMWH+/HR−) (154 ± 7 vs 128 ± 19, respectively, *p*=0.024). Only HR+ patients received intraoperative additional doses of UFH (150 U/kg), and there was no significant difference between Group 2 (LMWH−/HR+) and Group 4 (LMWH+/HR+).

Significant differences were found between the HR+ and HR− patients in terms of CPB duration and aortic clamp duration (94.2 ± 55.9 min vs 67.8 ± 26.0 min, *p*=0.033 and 51.52 ± 18.7 min vs 45.5 ± 17.9 min, *p*=0.022). We performed a power analysis to exclude underpowered outcomes. Variables such as LMWH, CPB time, and aortic clamp time achieved power more than 85%. Only 2 patients needed mediastinal re-exploration due to excessive bleeding, and both patients were in Group 4 (LMWH+/HR+).

Compared to all other groups, mortality was significantly higher in Group 4 (*n*=4) (LMWH+/HR+) (*p*=0.013).

## 4. Discussion

This current article shows the relation between preoperative usage of LMWH and HR. The definition of the causes and effects of HR are still controversial in cardiac surgery practice. There are very few studies examining the effects of preoperative LMWH therapy on HR during cardiac surgery [5, 13]. In this study, we specifically compared adult patients undergoing on-pump CABG surgery who received preoperative LMWH with a control group who did not receive LMWH before surgery. The overall incidence of intraoperative HR was 20.9% (*n*=29), which was similar to that of other studies [2, 3]. The incidence of intraoperative HR was observed significantly higher in the group with preoperative LMWH treatment. These results were similar to the previously published data of preoperative UFH treatment [14, 15]. LMWH and UFH both exert their effects through the same AT pathway. UFH leads to equivalent inactivation of factor X and factor IIa, while LMWH mostly leads to inactivation of factor X. Since both drugs act over AT pathway for anticoagulant effect, this may be the explanation of similar results [9, 10]. Most recent investigations provide evidence that there is a significant positive correlation between preoperative platelet count and intraoperative HR development [12, 16, 17]. However, there are contradictory results in the literature. Brinks et al. [18] demonstrated a relation between preoperative lower platelet counts in patients who developed intraoperative HR. Our results were also parallel to their findings. Although preoperative serum platelet levels were in the normal range in all groups, platelet levels were significantly lower in HR+ groups (Group 2 and Group 4). In contrast to our study, Brinks et al. [18] did not demonstrate any difference in platelet counts between groups with and without LMWH pretreatment and concluded that this was most likely due to the inadequate definition of HR. As mentioned before, the ACT response to UFH is complex and affected by different factors. Although AT pathway is the main mechanism for the anticoagulant effect of UFH, there are other factors, such as plasma protein binding, leukocyte, lactoferrin, and activated platelet count [3].

Dietrich and et al. [14] demonstrated that patients with intraoperative HR showed prolonged CPB and aortic clamp times. Results of the current study were also similar to these findings. However, there are also studies that show no effect of CPB and aortic clamp times on HR [7, 18]. These results are proof of an ongoing discussion.

ACT levels 5 minutes after the administration of protamine sulfate was also measured in this study. Kanbak et al. [19] found no significant difference between HR+ and HR– groups in terms of ACT levels after protamine. In this study, ACT levels after protamine in LMWH pretreated patients were higher, but the comparison was irrespective of HR presence. Unlike UFH, LMWH agents have longer half-lives [11]. Cavusoglu et al. [20] showed in their study that LMWH agents might have an effect on ACT levels.

There are intraoperative treatment strategies for HR, such as fresh frozen plasma (FFP) or AT administration. Because of its easy accessibility and lower prices, FFP is the most common therapy in current practice. In this study, it was found that Group 4 (LMWH+ / HR+) had received significantly higher amounts of intraoperative FFP (*p* < 0.001). Although this increased FFP requirement might be explained by the defect of an intraoperative bleeding profile of Group 4, there was no significant difference between the groups in terms of mediastinal re-exploration due to bleeding complications. However, there are many studies showing higher rates of mediastinal re-exploration in LMWH pretreated patients [7]. This might show the effectiveness of adequate intraoperative FFP therapy to prevent bleeding complications. Our mortality rate was 3.6% (*n*=5). Four out of five patients that died were in Group 4, and mortality rate was found significantly higher in Group 4 (HR+ / LMWH+) than other groups. Ranucci et al. [3] also showed significantly higher rates of mortality in HR groups, even though there was no difference in terms of patient demographics. However, in our study, LMWH pretreated patients had more critical coronary artery diseases, which can be one of the reasons to explain higher rates of mortality.

Besides the findings of this study, there are some limitations. This study was conducted in a single institution with a limited sample size. Our study is a retrospective design study, laboratory tests for congenital or acquired AT deficiencies are not in our routine preoperative screening, and we could not identify the undiagnosed AT deficiencies. Relatively small sample size might have precluded us to detect minor differences between the groups. The preoperative administration of enoxaparin was not controlled, and although there were no significant differences between the groups, effects of preoperative usage of ASA and clopidogrel on HR were not investigated. Progression of HR is strongly related to the dose of UFH to achieve a specific target ACT. Because of this strong relationship, our results may be estimated to higher target ACTs, and this might have affected our results. We found the longer duration of ICU and hospital stay in HR+ groups. However, further prospective trials with homogenous groups are needed for the explanation of this relation between HR and postoperative outcome.

In conclusion, preoperative LMWH treatment may cause HR in patients undergoing on-pump CABG surgery. In general, practice to overcome HR during on-pump cardiac surgery higher amounts of UFH is used. Inevitably, using higher amounts of UFH may have effects on early postoperative outcome such as bleeding. Therefore, detection of preoperative HR should be a warning for postoperative events. Corrective and preventive management with close follow-up should be performed in this group of patients. Further prospective clinical research studies in larger patient populations are necessary to encourage our results.

## Figures and Tables

**Table 1:** Patient demographics, clinical characteristics, and operative data.

	HR− (*n*=110)		HR+ (*n*=29)		*P* value
	Group 1 LMWH− (*n*=55)	Group 3 LMWH+ (*n*=55)	Group 2 LMWH− (*n*=5)	Group 4 LMWH+ (*n*=24)	
Age (y)	66 ± 10	63 ± 9	63 ± 11	65 ± 11	0.463
Gender (F) (*n*)	22	20	2	6	0.658
Weight (kg)	77 ± 12	80 ± 12	77 ± 7	80 ± 16	0.598
Preoperative platelet count (×1000 *μ*L)	249 ± 74	275 ± 85	168 ± 70	197 ± 75	<0.001^*∗*^
*Intraoperative patients' data*					
CPB time (min)	67.8 ± 26.0		94.2 ± 55.9		0.033^*α*^
Aortic clamp time (min)	45.5 ± 17.9		51.52 ± 18.7		0.022^*α*^
Basal ACT (sec)	126 ± 16	125 ± 15	119 ± 18	122 ± 15	0.654
Postheparin ACT (sec)	633.58 ± 205.1		450.69 ± 133.2		0.001^*α*^
Postprotamine ACT (sec)	133 ± 22	154 ± 7	128 ± 19	133 ± 25	0.034+
Intraoperative FFP used (units)	2.1 ± 0.9	3.8 ± 1.8	2.1 ± 0.9	4.5 ± 1.8	<0.001^x^
Intraoperative thrombocyte used (units)	0.3 ± 1.3	1.0 ± 2.2	0.3 ± 1.5	2.2 ± 3.1	0.001^x^
Reexploration (*n*)	0	0	0	2	0.980
Mortality (*n*)	1	0	0	4	0.013^y^

Data are presented as mean ± SD. LMWH, low-molecular-weight heparin; HR, heparin resistance; *n*, number; y, years; F, female; kg, kilograms; CPB, cardiopulmonary bypass; min, minutes; ACT, active coagulation time; sec, seconds; FFP, fresh frozen plasma. ^*∗*^Significant difference was between Groups 2 and 3. ^*α*^Significant difference was between groups HR+ and HR−. ^x^Significant difference was between Groups 1 and 4 and Groups 2 and 4. ^y^Significant difference was between Groups 2 and 4 and Groups 3 and 4.

## Data Availability

The datasets generated and/or analyzed during the current study are available from the corresponding author on reasonable request.
